# Wind tunnel measurement dataset of turbulent flow over two-dimensional ridges and three-dimensional hills with smooth and rough surfaces

**DOI:** 10.1016/j.dib.2025.112260

**Published:** 2025-11-12

**Authors:** Takeshi Ishihara, Tong Zhou

**Affiliations:** Department of Civil Engineering, School of Engineering, The University of Tokyo, Tokyo, 113-8656, Japan

**Keywords:** Wind tunnel experiment, Ridge, Hill, Surface roughness, Mean velocity, Turbulent statistics

## Abstract

This paper presents a high-spatiotemporal-resolution dataset of turbulent flows over two-dimensional (2D) ridges and three-dimensional (3D) hills with smooth and rough surfaces measured in a boundary-layer wind tunnel. This dataset extends the experimental measurements reported in the research article entitled “A wind tunnel study of turbulent flow over a three-dimensional steep hill” by Ishihara et al. (1999). In addition to the original 3D smooth surface case, new measurements on 2D smooth and rough ridges and a 3D rough hill are conducted to clarify the effects of surface roughness and topography on the hill-induced flow field. The three velocity components on multiple representative horizontal and vertical planes around the hilly terrains were measured under different surface roughness conditions. Mean and fluctuating velocity profiles in the wake region are presented along with details of the measurement and data acquisition procedures. This database provides methodological guidance for future wind tunnel studies over complex terrain, including probe arrangement, data acquisition, and experimental design to capture complex mountainous wind fields. Furthermore, it provides a benchmark for validating computational fluid dynamics (CFD) simulations, improving turbulence models, and assessing wind resources in mountainous regions.

Specifications Table

**Table:** 

Subject	Engineering & Materials science
Specific subject area	Wind engineering, Wind environment, Wind energy
Type of data	Table, Image, Figure. Raw, Analyzed.
Data collection	Constant-temperature hot-wire anemometers (DANTEC 56C01 and 56C17) and X-wire probes (DANTEC; 55R53 and 55R54) were used to measure the three velocity components in the undisturbed boundary layer. Split-fiber probes (DANTEC 55R55 and 55R57) were used to measure the three velocity components over the 2D ridges and 3D hills. A pitot tube was employed to calibrate the X-wires probe measurement in the free stream. A Pitot tube was employed to calibrate the split-fiber probe measurements, and a Draft master (Kanomax model 6311) was used when the free-stream velocity was less than 1 m/s. A manometer was employed to measure the pressure difference. Two boundary layer flow are considered: a smooth surface (u_*_ = 0.21 m/s, z_0_ = 0.01 m) and a rough surface (u_*_ = 0.32 m/s, z_0_ = 0.3 m), both at full scale with a scale ratio of 1/1000. Four cases are considered: a 2D smooth ridge (Case 2Ds), a 2D rough ridge (Case 2Dr), a 3D smooth hill (Case 3Ds) and a 3D rough hill (Case 3Dr). The global Reynolds numbers based on the hill height h and reference velocity Uh are 1.2 × 10^4^ and 1 × 10^4^ for smooth and rough cases, respectively. Furthermore, the surface Reynold numbers based on the friction velocity u_*_ and roughness length z_0_ are 0.14 and 6.4 for smooth and rough cases, respectively. The sampling time and sampling frequency for the velocity measurements were 60 s and 1000 Hz, respectively.
Data source location	Institution: The University of Tokyo City/Town/Region: Tokyo/Bunkyo-ku/Hongo Country: Japan
Data accessibility	Repository name: Mendeley Data Data identification number: 10.17632/dc7f82pc49.2 Direct URL to data: https://data.mendeley.com/datasets/dc7f82pc49/2
Related research article	T. Ishihara, K. Hibi, and S. Oikawa, “A wind tunnel study of turbulent flow over a three-dimensional steep hill,” J. Wind Eng. Ind. Aerodyn., 83(1–3), 95–107, 1999: https://doi.org/10.1016/S0167–6105(99)00,064–1

## 1 Value of the Data

• The dataset provides a high-spatiotemporal-resolution database for quantifying the turbulent flows over 2D ridges and 3D hills with smooth and rough surfaces.
• The dataset serves as a valuable reference for researchers in the field of wind engineering, wind environment and wind energy.
• The dataset contributes methodological insights into future wind tunnel research in hilly areas, including probe arrangement, data acquisition, and experimental design for characterizing complex wind fields in mountainous regions [[2], [3], [4], [5], [6], [7]].
• The dataset serves as a benchmark for performance evaluation of different CFD configurations, including grid resolution, turbulence models, inflow turbulence generation methods, subgrid-scale models and numerical schemes [[8], [9], [10], [11], [12], [13], [14], [15], [16], [17], [18], [19]].

## 2 Background

Advancing the understanding of flow characteristics over hilly terrains within the atmospheric boundary layer is essential for diverse engineering applications, including structural safety, wind energy exploitation, pollutant dispersion, agricultural and forestry protection, and aviation safety.

The present dataset provides high-resolution quantitative information on mean flow and turbulence statistics over representative hilly terrains, obtained using split-fiber probes. It complements the original research paper “A wind tunnel study of turbulent flow over a three-dimensional steep hill” [1] and adds value by establishing a benchmark reference for complex-terrain flows. Additionally, the present dataset systematically investigates the flow field over 2D ridges and 3D hills with different surface roughness, filling the gap in previous experimental investigations that mainly focused on the influence of a single factor. Moreover, the dataset can be directly applied to the comprehensive validation of computational fluid dynamics (CFD) simulations, the development of turbulence models, and is broadly reusable for wind resources assessment, environmental dispersion evaluation and structural safety analysis in mountainous areas.

## 3 Data Description

The dataset presented in this paper are described in the following:

**Approaching flow characteristics over flat smooth surface (supplementary file Table 1):**

The vertical profiles of mean and fluctuating velocity components for the undisturbed boundary layer over flat smooth surface, measured at the center of the turntable are shown in this file.

**Approaching flow characteristics over flat rough surface (supplementary file Table 2):**

The vertical profiles of mean and fluctuating velocity components for the undisturbed boundary layer over flat rough surface, measured at the center of the turntable are shown in this file.

For clarity, a summary of experimental measurements of the approaching flows is illustrated in Table 1 as follows:

**Table 1:** Measurement details of the approaching flows over flat smooth surface.

Num.	x (mm)	y (mm)	z (mm)	U (m/s)	W (m/s)	$\sigma_{u}$ (m/s)	$\sigma_{v}$ (m/s)	$\sigma_{w}\;$(m/s)
1∼18	0	0	5, 7, 10, 15, 20, 30, 40, 45, 50, 60, 80, 100, 120, 160, 200, 240, 280, 360	…	…	…	…	…

**Turbulent statistics over the vertical plane for Case 2Ds (supplementary file Table 3):**

This table provides the vertical distributions of mean and fluctuating velocity components for turbulent flow over the 2D smooth ridge, measured in the vertical center plane (*y* = 0).

**Turbulent statistics over the vertical plane for Case 2Dr (supplementary file Table 4):**

This table provides the vertical distributions of mean and fluctuating velocity components for turbulent flow over the 2D rough ridge, measured in the vertical center plane (*y* = 0).

**Turbulent statistics over the vertical plane for Case 3Ds (supplementary file Table 5):**

This table provides the vertical distributions of mean and fluctuating velocity components for turbulent flow over the 3D smooth hill, measured in the vertical center plane (*y* = 0).

**Turbulent statistics over the vertical plane for Case 3Dr (supplementary file Table 6):**

This table provides the vertical distributions of mean and fluctuating velocity components for turbulent flow over the 3D rough hill, measured in the vertical center plane (*y* = 0).

For clarity, a summary of experimental measurements for vertical profiles of turbulent statistics over a 3D smooth hill is illustrated in Table 2 as follows:

**Table 2:** Measurement details of vertical profiles of turbulent statistics over a 2D smooth ridge.

Num.	*x* (mm)	*y* (mm)	*z*′ = *z*–*z_s_* (mm)	*U* (m/s)	*W* (m/s)	*σ_u_* (m/s)	*σ_v_* (m/s)	*σ_w_* (m/s)
1∼18	-150	0	5, 7, 10, 15, 20, 30, 40, 45, 50, 60, 80, 100, 120, 160, 200, 240, 280, 360	…	…	…	…	…
19∼36	-100	0	5, 7, 10, 15, 20, 30, 40, 45, 50, 60, 80, 100, 120, 160, 200, 240, 280, 360	…	…	…	…	…
37∼54	-50	0	5, 7, 10, 15, 20, 30, 40, 45, 50, 60, 80, 100, 120, 160, 200, 240, 280, 360	…	…	…	…	…
55∼72	0	0	5, 7, 10, 15, 20, 30, 40, 45, 50, 60, 80, 100, 120, 160, 200, 240, 280, 360	…	…	…	…	…
73∼90	50	0	5, 7, 10, 15, 20, 30, 40, 45, 50, 60, 80, 100, 120, 160, 200, 240, 280, 360	…	…	…	…	…
91∼108	100	0	5, 7, 10, 15, 20, 30, 40, 45, 50, 60, 80, 100, 120, 160, 200, 240, 280, 360	…	…	…	…	…
109∼126	150	0	5, 7, 10, 15, 20, 30, 40, 45, 50, 60, 80, 100, 120, 160, 200, 240, 280, 360	…	…	…	…	…
127∼144	200	0	5, 7, 10, 15, 20, 30, 40, 45, 50, 60, 80, 100, 120, 160, 200, 240, 280, 360	…	…	…	…	…
145∼162	250	0	5, 7, 10, 15, 20, 30, 40, 45, 50, 60, 80, 100, 120, 160, 200, 240, 280, 360	…	…	…	…	…

**Turbulent statistics over the horizontal planes for Case 3Ds (supplementary files Table 7):**

This table provides the vertical distributions of mean and fluctuating velocity components for turbulent flow over the 3D smooth hill, measured in the horizontal center plane (*z/h* = 0.125 and 1).

**Turbulent statistics over the horizontal planes for Case 3Dr (supplementary files Table 8):**

This table provides the vertical distributions of mean and fluctuating velocity components for turbulent flow over the 3D rough hill, measured in the horizontal center plane (*z/h* = 0.25 and 1).

For clarity, a summary of experimental measurements for horizontal profiles of turbulent statistics over a 3D smooth hill is illustrated in Table 3 as follows:

**Table 3:** Measurement details of horizontal profiles of turbulent statistics over a 3D smooth hill.

Num.	*x* (mm)	*y* (mm)	*z* (mm)	*U* (m/s)	*V* (m/s)	*σ_u_* (m/s)	*σ_v_* (m/s)	*σ_w_* (m/s)
1∼13	150	-200, 150, -100, -90, -80, -70, -60, -50, -40, -30, -20, -10, 0	5	…	…	…	…	…
14∼26	150	-200, 150, -100, -90, -80, -70, -60, -50, -40, -30, -20, -10, 0	40	…	…	…	…	…

## 4 Experimental Design, Materials and Methods

### 4.1 Measurement setup

**Fig. 1:**
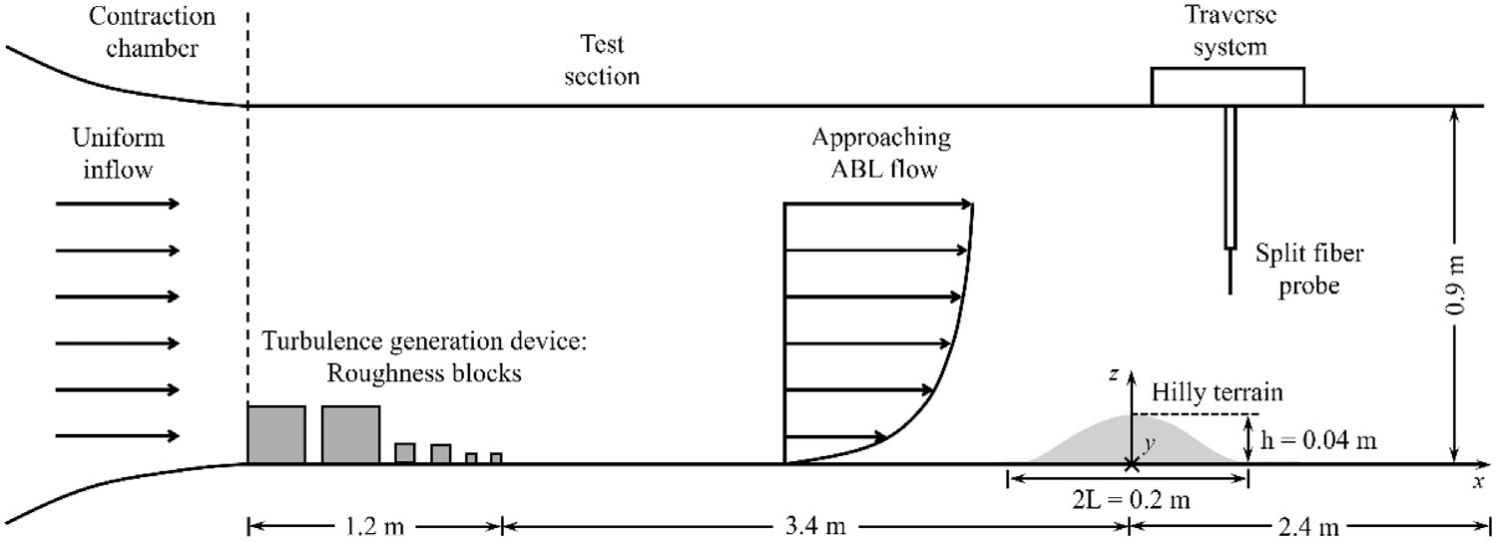
Configuration of the wind tunnel experiment [1].

The wind tunnel experiments were performed in the boundary layer wind tunnel at Shimizu Corporation. The closed-circuit tunnel has a test section of 7.0 m in length, 1.1 m in width, and 0.9 m in height. As shown in Fig. 1, a uniform flow with a mean velocity of 5.9 m/s and a turbulence intensity of 0.5 % was introduced into the test section through the contraction chamber.

**Fig. 2:**
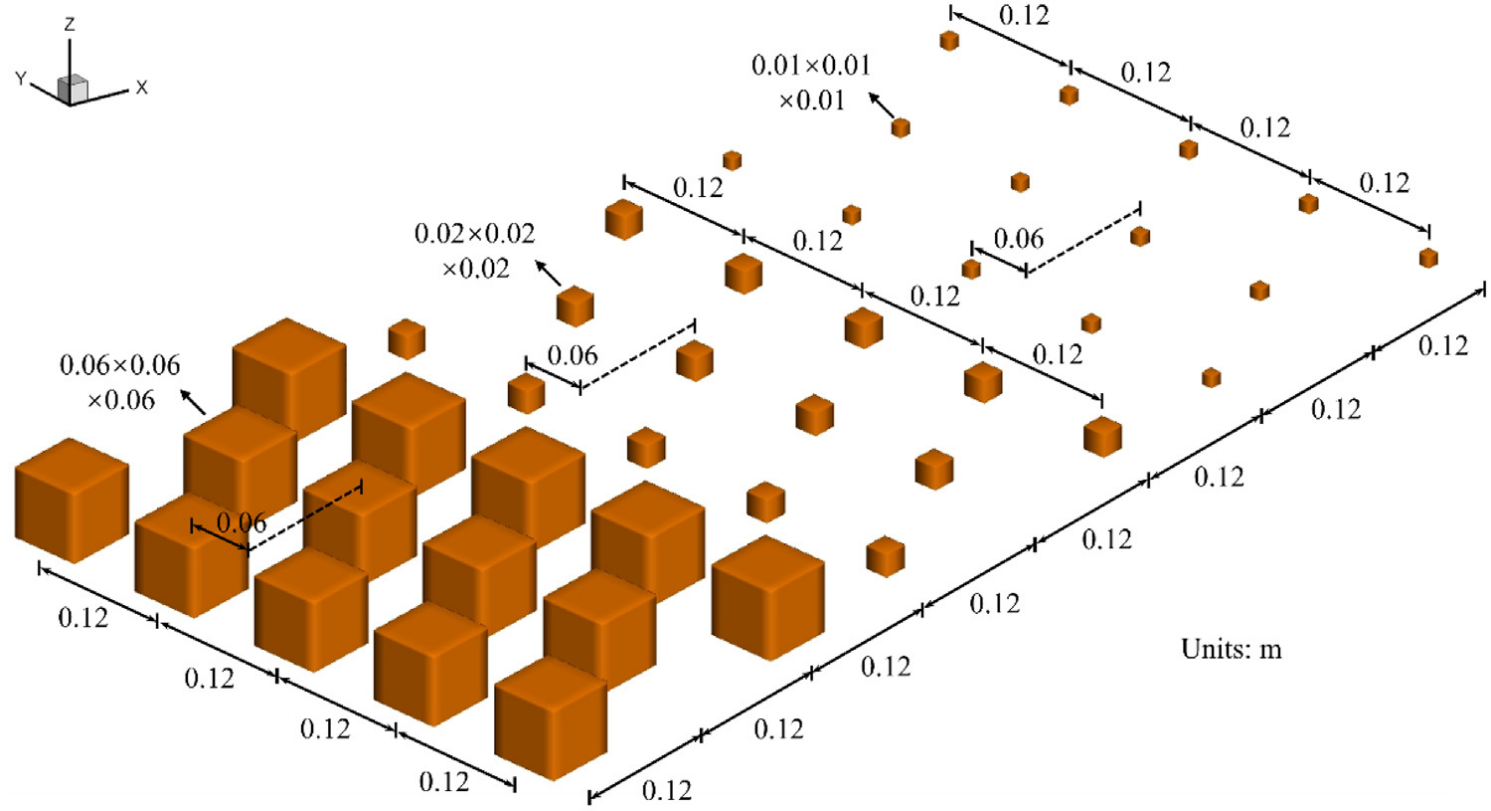
Detail of roughness block arrangement.

The turbulent boundary layer was generated by an array of cubic roughness blocks placed on the test-section floor over a longitudinal distance of 1.2 m downstream of the contraction outlet. As illustrated in Fig. 2, the roughness blocks consisted of 0.06 m high cubes followed by 0.02 m and 0.01 m cubes in a staggered pattern. The areal densities of these roughness blocks were 25 %, 2.8 % and 0.7 %, respectively. Each group comprised three rows of blocks, each 0.4 m long in the streamwise direction.

**Fig. 3:**
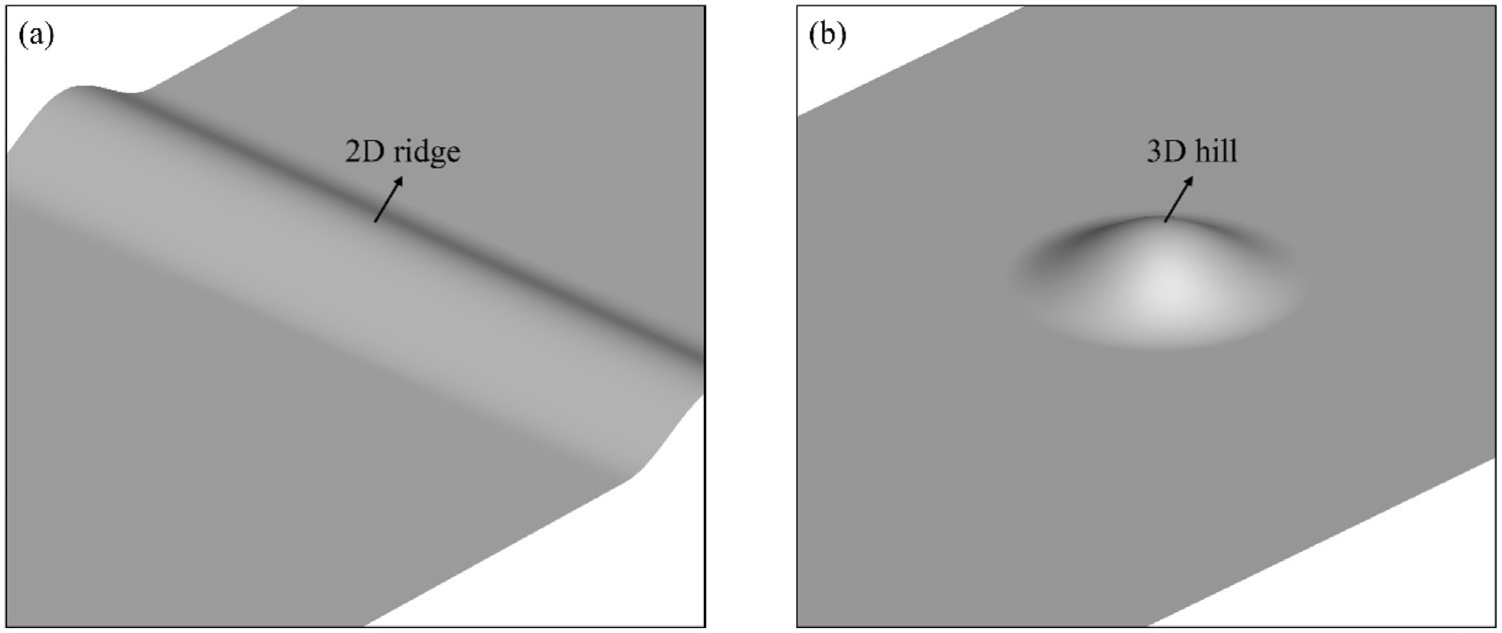
Geometric shape of hilly terrains: (a) 2D ridge, (b) 3D hill.

Two types of hilly terrains were considered in the wind tunnel experiments: (a) 2D ridge and (b) 3D hill, as depicted in Fig.3. The terrain profiles of 2D ridge and 3D hill are defined by the following cosine-based functions:

(1) $$z_{s}\left(x,y\right)=hcos^{2}\pi x/2L$$

(2) $$z_{s}\left(x,y\right)=hcos^{2}\pi {\left(x^{2}+y^{2}\right)}^{\frac{1}{2}}/2L$$

where h is the terrain height and L is the half-length of the ridge or hill. The maximum terrain gradient is approximately 32°.

**Fig. 4:**
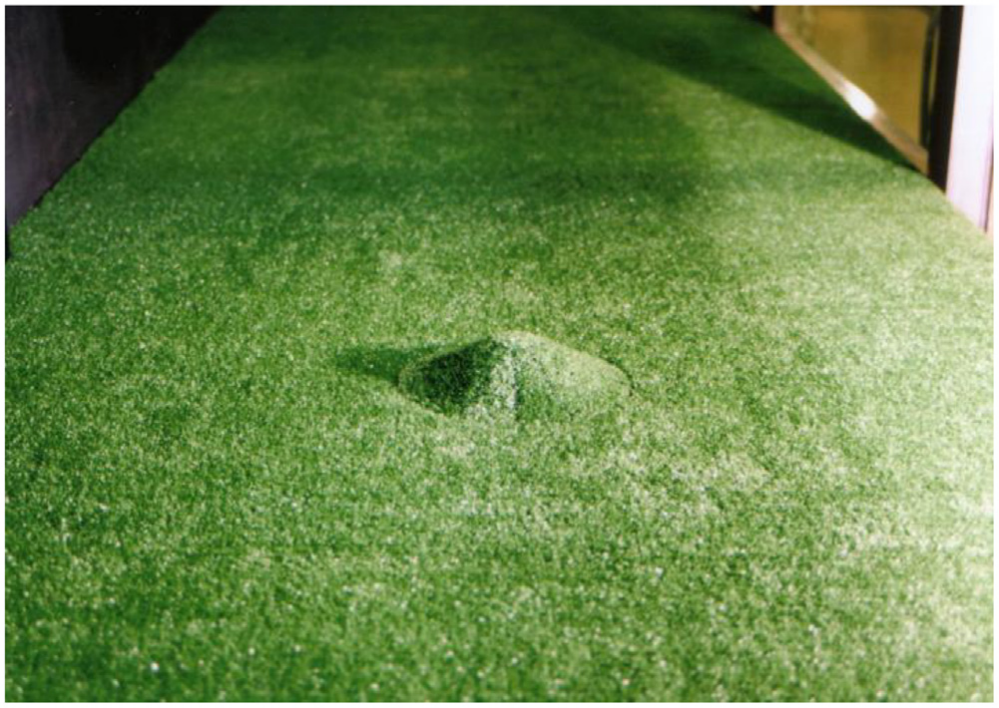
Photo of the 3D rough hill.

Two types of surface roughness conditions were considered: a smooth surface (z_0_ = 0.01 m in full scale) and a rough surface (z_0_ = 0.3 m in full scale). For the rough surface, the terrain surface was uniformly covered with artificial grass with a height of 0.005 m, as shown in Fig. 4.

Approaching boundary layer velocities were measured using constant-temperature hot-wire anemometers (DANTEC 56C01, 56C17) equipped with X-wire probes (55R53, 55R54). The X-wires were calibrated against a Pitot tube in the free stream using the polynomial relationship *U* = aE^3^ + bE^2^ + cE + *d*, where U is the velocity and E is the voltage. The calibration coefficients a, b, c and d were obtained by least-squares fitting, with deviations less than 1 %.

### 4.2 Calibration and sampling methods

**Fig. 5:**
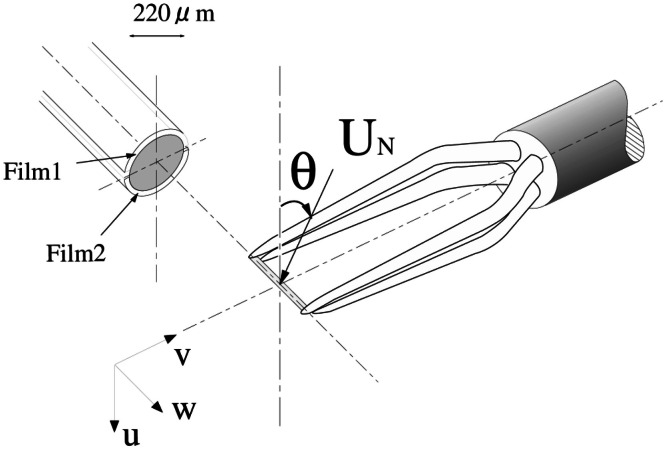
Schematic of the 55R55 split-fiber probe [1].

Since the accuracy of X-wire probes decreases when turbulence intensity exceeds 0.3 [20], all velocity measurements around the hill were performed with split-fiber probes. The 55R55 straight probe, with its split plane normal to the freestream, was used to detect reverse flows and measure the longitudinal velocity. In addition, the 55R57 90° probe, with the split plane parallel to the free stream, was used to measure the lateral and vertical velocities. To ensure accurate measurements, the overheat ratios of the two films shown in Fig. 5 were closely matched.

To measure one velocity component defined in Fig. 5, two calibration functions were implemented for the normal velocity magnitude U_N_ and the pitch angle θ, respectively.

(3) $$U_{N}=f\left(E_{1}+E_{2}\right)$$

(4) $$\theta =\left\{ \begin{array}{cc} cos^{-1}\left(\Delta E/\Delta E_{0}\right), & E_{1}\geq E_{2}\\ cos^{-1}\left(\Delta E/\left|\Delta E_{180}\right|\right), & E_{1}<E_{2} \end{array}\right.$$

(5) $$\Delta E=g\left(\theta \right)\Delta E_{max}\left(U_{N}\right)$$

where E_1_ and E_2_ are the voltages from the two films, f is a cubic spline function, ∆E is the output difference between the films, g is the empirical calibration function, ∆E_max_ is the maximum output difference, ∆E_0_ and ∆E_180_ are the differences corresponding to the pitch angle of 0° and 180° Calibrations were performed by rotating the probe in a smooth, uniform flow.

The probe response was validated by comparing the measured longitudinal velocity spectra with those from a single hot-wire probe at U_∞_ = 2 m/s and 6 m/s, showing good agreement in the frequency range below 1 kHz. Furthermore, the signals were sampled at 2 kHz and low-pass filtered at 1 kHz. A sampling duration of 60 s was used for mean velocity and turbulent statistics.

**Fig. 6:**
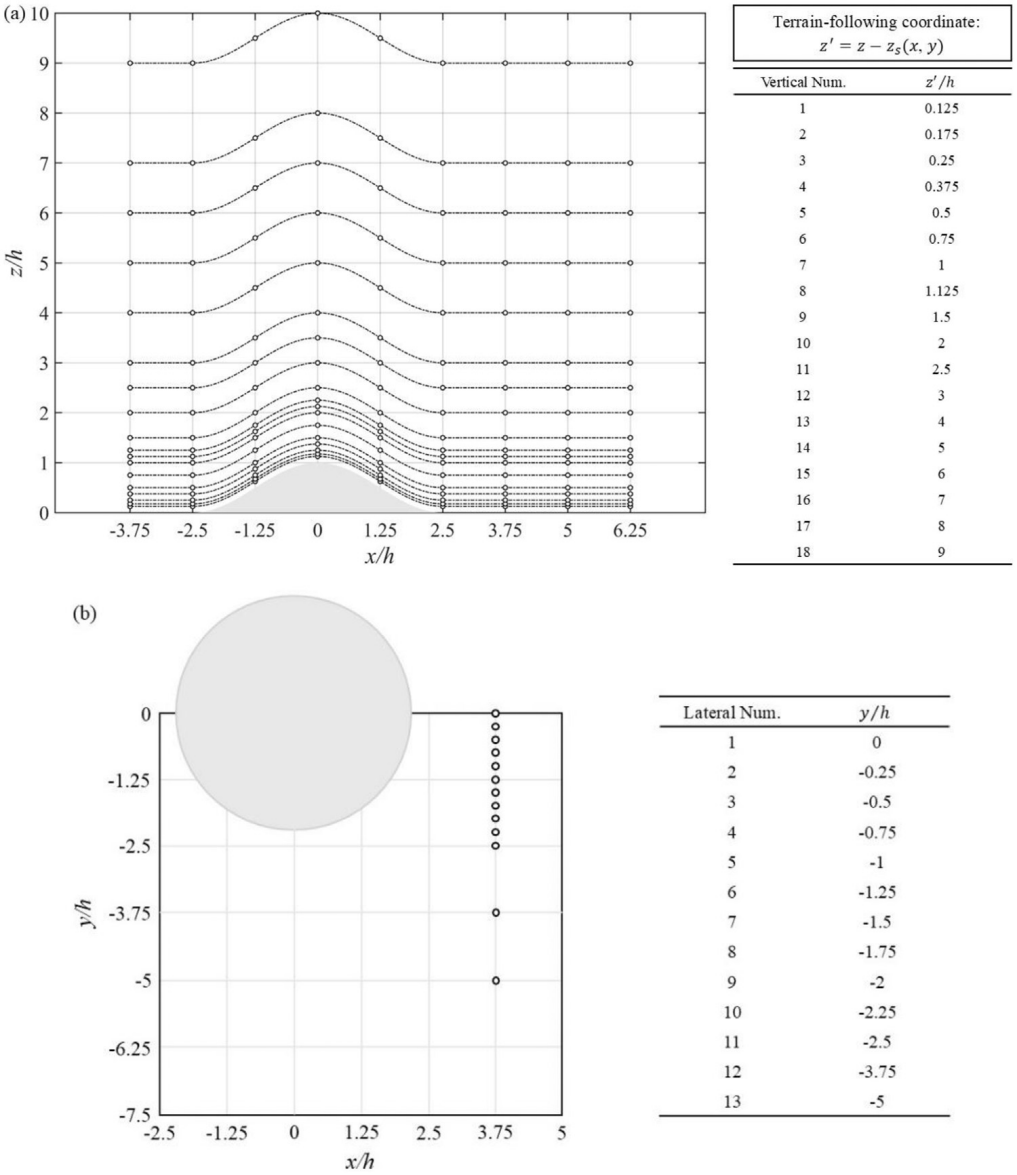
Layout of measurement points: (a) vertical and (b) horizontal planes.

The wind velocity measurement locations around the hilly terrains are presented in Fig. 6.

## Limitations

The wind tunnel experiments are performed over idealized terrain and under simplified atmospheric inflow conditions. However, real terrains are often characterized by variable slope, complex shape and heterogeneous surface roughness, resulting in more complex local flow structures. Furthermore, the inflow does not consider the effects of Coriolis force and thermal stratification, which may introduce uncertainties in generalizing the results to atmospheric flows over hilly terrain. Moreover, although split-fiber probes provide high measurement accuracy, the experimental dataset is inherently subject to limitations associated with the finite number of sampling points. In particular, the number of horizontal measurement planes is restricted, which constrains the spatial representation of the turbulent flow field around hilly terrain.

## Ethics Statement

The authors have read and followed the ethical requirements for publication in Data in Brief and confirmed that the current work does not involve human subjects, animal experiments, or any data collected from social media platforms.

## Credit Author Statement

**Takeshi Ishihara:** Conceptualization, Methodology, Investigation, Supervision, Funding acquisition, Writing – review & editing; **Tong Zhou:** Formal analysis, Validation, Visualization, Data Curation, Writing – original draft.

## Data Availability

Mendeley DataExperimental dataset of turbulent boundary layer flows over simplified hilly terrains (Original data).

## Appendix Supplementary materials

Image, application 1

Image, application 2

Image, application 3

Image, application 4

Image, application 5

Image, application 6

Image, application 7

Image, application 8
